# Contrasting Pathogenic Mechanisms in Acute and Subacute Gallbladder Volvulus: Analysis of Two Cases with Different Degrees of Torsion

**DOI:** 10.7759/cureus.75444

**Published:** 2024-12-10

**Authors:** Yuka Kawano, Yujo Kawashita, Shintaro Hirayama, Takashi Ueda, Masaki Tateishi, Junzo Yamaguchi, Sousei Abe, Yasuo Washida

**Affiliations:** 1 Surgery, Fukuoka Seisyukai Hospital, Fukuoka, JPN; 2 Radiology, Fukuoka Seisyukai Hospital, Fukuoka, JPN

**Keywords:** cholecystectomy laparoscopic, cholecystitis diagnosis, gallbladder volvulus, recurrent cholecystitis, wandering gallbladder

## Abstract

Gallbladder volvulus is a rare but potentially severe condition that requires urgent surgical intervention. This report presents two contrasting cases of gallbladder volvulus with distinct pathogenic mechanisms and degrees of torsion. The first case involves a 97-year-old female who presented with acute symptoms and 270° torsion, leading to complete gallbladder necrosis (Gross type II). The second case involves a 52-year-old male with a subacute presentation and 90° torsion, resulting in incomplete necrosis (Gross type I). Both cases underwent successful laparoscopic cholecystectomy, completed in 69 and 59 minutes, respectively. Analysis of these cases reveals that the degree of torsion correlates with the acuity of presentation and the extent of tissue ischemia, which influences the timing of surgical intervention. This study underscores the importance of early recognition and prompt surgical management in gallbladder volvulus, regardless of presentation pattern. Although gallbladder volvulus predominantly affects elderly females, it can occur across different age groups and genders, highlighting the need for increased clinical awareness to achieve optimal outcomes.

## Introduction

Gallbladder volvulus, first described by Wendel in 1898, represents a rare but potentially life-threatening surgical emergency characterized by the rotation of the gallbladder around its mesentery [1]. This condition predominantly affects elderly females, though cases across various demographic groups have been reported [2]. The pathophysiology generally involves a floating gallbladder with minimal hepatic attachment, allowing for abnormal mobility and potential torsion [3].

The clinical significance of gallbladder volvulus lies in its potential for rapid progression to ischemic changes and subsequent necrosis, necessitating urgent surgical intervention for optimal outcomes [4]. Despite technological advances in medical imaging, preoperative diagnosis remains challenging due to the variable and often nonspecific nature of presenting symptoms [5]. This diagnostic challenge is further complicated by the condition's relative rarity.

In this report, we present two contrasting cases of gallbladder volvulus with distinct pathogenic mechanisms, highlighting the importance of understanding this rare but potentially severe condition. Through detailed analysis of these cases, we aim to contribute to the growing body of knowledge regarding optimal diagnostic approaches and management strategies for this challenging surgical entity.

## Case presentation

Case 1

A 97-year-old female presented to the emergency department with vomiting and epigastric pain. On examination, tenderness was noted in the epigastric region without evidence of jaundice. Laboratory test results are summarized in Table 1.

**Table 1:** Laboratory test results in Case1 TP: Total Protein, Alb: Albumin, T-Bil: Total Bilirubin, AST: Aspartate Aminotransferase, ALT: Alanine Aminotransferase, γ-GTP: Gamma-Glutamyl Transferase, ALP: Alkaline Phosphatase, BUN: Blood Urea Nitrogen, Cre: Creatinine, CRP: C-Reactive Protein, WBC: White Blood Cells, %Neut: Percentage of Neutrophils, %Lymph: Percentage of Lymphocytes, %Mono: Percentage of Monocytes, %Eosio: Percentage of Eosinophils, %Baso: Percentage of Basophils, RBC: Red Blood Cells, Hb: Hemoglobin, Ht: Hematocrit, Plt: Platelets

Laboratory parameters	Case 1 values	Normal range
TP	9.0 g/dL	6.7-8.3 g/dL
Alb	3.4 g/dL	4.0-5.0 g/dL
T-Bil	0.79 mg/dL	0.30-1.20 mg/dL
AST	53 U/L	13-33 U/L
ALT	16 U/L	6-30 U/L
γ-GTP	20 U/L	10-47 U/L
ALP	122 U/L	38-113 U/L
BUN	10.0 mg/dL	8.0-22.0 mg/dL
Cre	0.42 mg/dL	0.60-1.10 mg/dL
Sodium	132 mEq/L	138-146 mEq/L
Potassium	4.4 mEq/L	3.6-4.9 mEq/L
Chloride	91 mEq/L	99-109 mEq/L
CRP	0.14 mg/dL	0.00-0.30 mg/dL
WBC	7,003 /μL	3,300-9,000 /μL
%Neut	81.1 %	44.0-72.0 %
%Lymph	11.6 %	18.0-59.0 %
%Mono	7.0 %	0.0-12.0 %
%Eosio	0.0 %	0.0-10.0 %
%Baso	0.3 %	0.0-3.0 %
RBC	4.04 ×10^6^/μL	4.30-5.70 ×10^6^/μL
Hb	13.2 g/dL	13.5-17.5 g/dL
Ht	40.4 %	42.0-53.0 %
Plt	1.79 ×10^5^/μL	1.20-3.50 ×10^5^/μL

**Figure 1:**
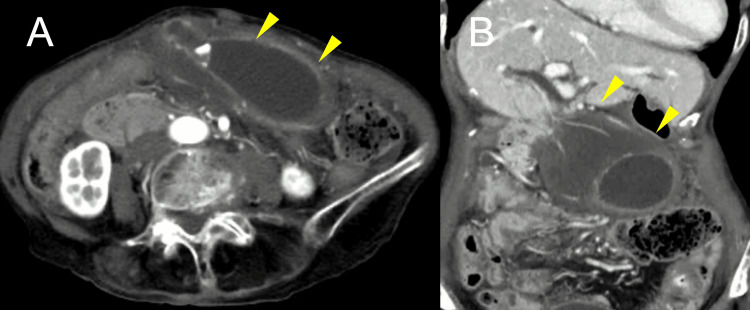
Radiological findings of gallbladder volvulus in Case 1 (A) Axial CT image shows medial displacement of the gallbladder with significant wall edema (yellow arrows). (B) Coronal CT image highlights the gallbladder’s medial displacement and edema (yellow arrows).

CT imaging revealed medial displacement of the gallbladder with significant wall edema (Figure 1). 

**Figure 2:**
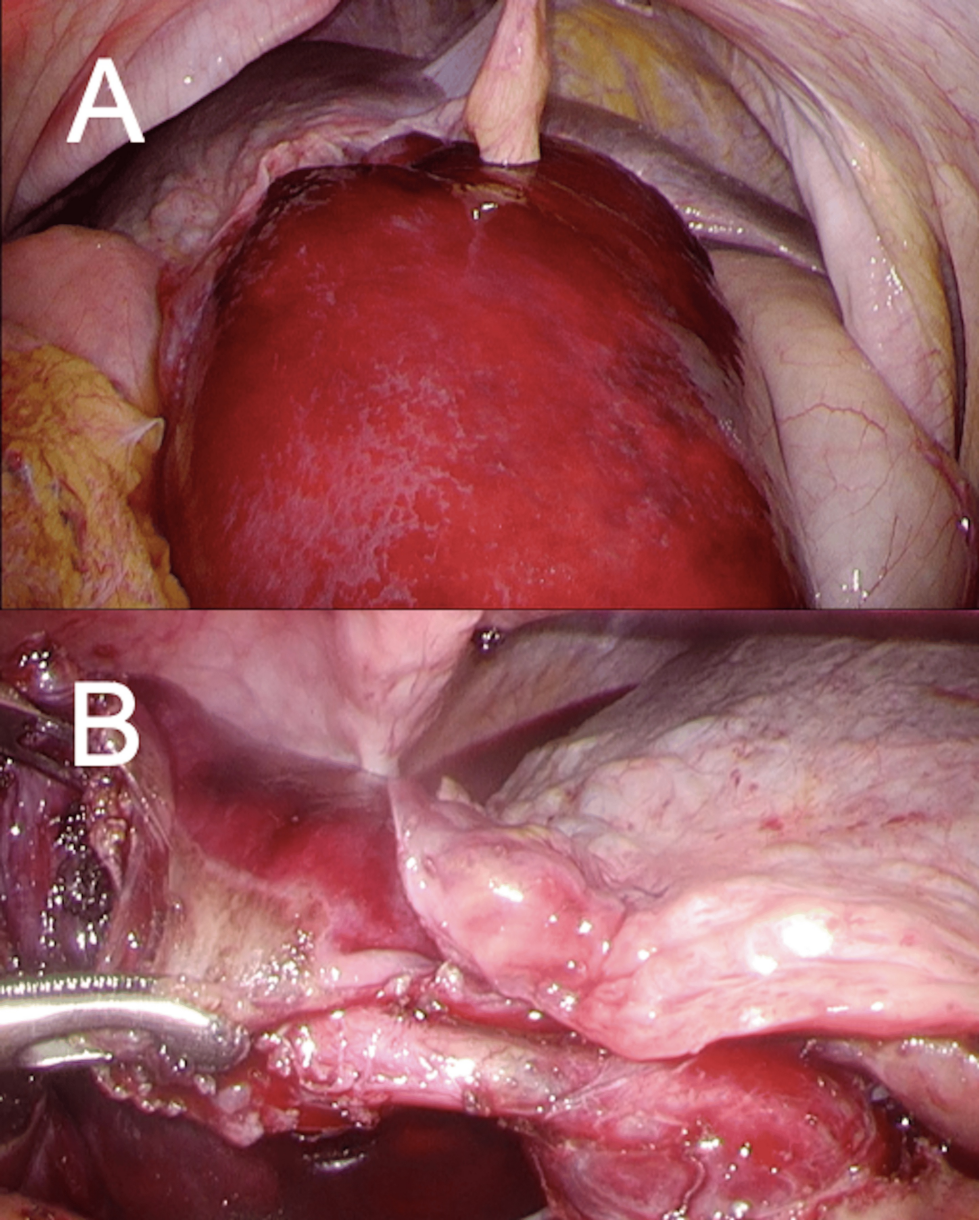
Intraoperative laparoscopic findings of Case 1 (A) Initial laparoscopic view shows a markedly enlarged, dark red gallbladder with severe ischemic changes. (B) Close-up view of the twisted gallbladder demonstrates 270°torsion.

Laparoscopic exploration demonstrated a markedly enlarged, dark red gallbladder with 270° torsion, classified as Gross type II with complete necrosis (Figure 2).

Laparoscopic cholecystectomy was completed in 69 minutes. The patient had an uneventful postoperative course and was discharged on postoperative day 5 without complications.

Case 2

A 52-year-old male presented with a one-month history of intermittent right hypochondrial pain, which worsened with specific movements, such as bending forward or reaching while driving. Initially mild, the symptoms escalated to severe pain, prompting his visit to the emergency department. Laboratory test results are summarized in Table 2.

**Table 2:** Laboratory test results in Case 2. TP: Total Protein, Alb: Albumin, T-Bil: Total Bilirubin, AST: Aspartate Aminotransferase, ALT: Alanine Aminotransferase, γ-GTP: Gamma-Glutamyl Transferase, ALP: Alkaline Phosphatase, BUN: Blood Urea Nitrogen, Cre: Creatinine, CRP: C-Reactive Protein, WBC: White Blood Cells, %Neut: Percentage of Neutrophils, %Lymph: Percentage of Lymphocytes, %Mono: Percentage of Monocytes, %Eosio: Percentage of Eosinophils, %Baso: Percentage of Basophils, RBC: Red Blood Cells, Hb: Hemoglobin, Ht: Hematocrit, Plt: Platelets

Laboratory parameters	Case 2 values	Normal range
TP	7.1 g/dL	6.7-8.3 g/dL
Alb	3.9 g/dL	4.0-5.0 g/dL
T-Bil	0.54 mg/dL	0.30-1.20 mg/dL
AST	15 U/L	13-33 U/L
ALT	10 U/L	6-30 U/L
γ-GTP	51 U/L	10-47 U/L
ALP	87 U/L	38-113 U/L
BUN	7.0 mg/dL	8.0-22.0 mg/dL
Cre	0.84 mg/dL	0.60-1.10 mg/dL
Sodium	139 mEq/L	138-146 mEq/L
Potassium	4.7 mEq/L	3.6-4.9 mEq/L
Chloride	105 mEq/L	99-109 mEq/L
CRP	0.43 mg/dL	0.00-0.30 mg/dL
WBC	8,736 /μL	3,300-9,000 /μL
%Neut	68.9 %	44.0-72.0 %
%Lymph	21.1 %	18.0-59.0 %
%Mono	8.1 %	0.0-12.0 %
%Eosio	1.3 %	0.0-10.0 %
%Baso	0.6 %	0.0-3.0 %
RBC	4.50 ×10^6^/μL	4.30-5.70 ×10^6^/μL
Hb	14.7 g/dL	13.5-17.5 g/dL
Ht	43.5 %	42.0-53.0 %
Plt	2.77 ×10^5^/μL	1.20-3.50 ×10^5^/μL

**Figure 3:**
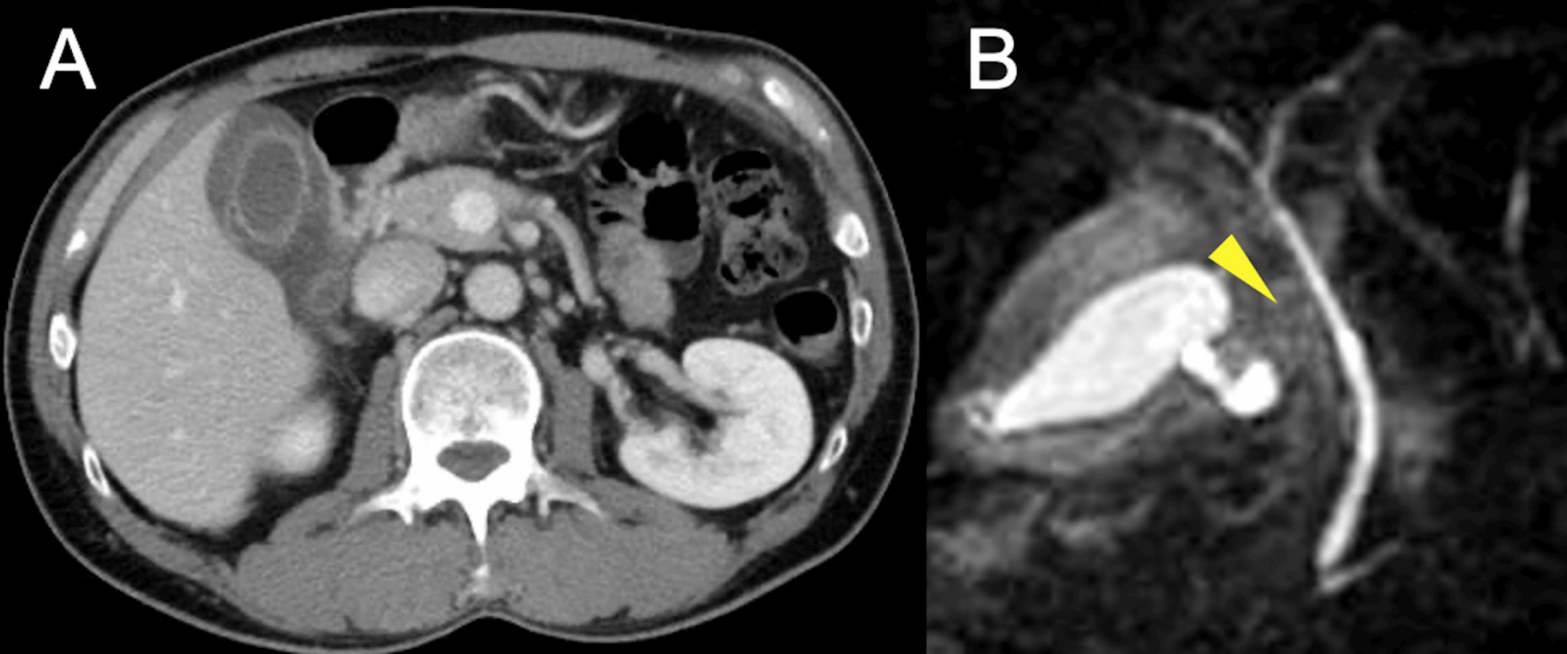
Radiological findings of gallbladder volvulus in Case 2 (A) CT image shows significant thickening and edema of the gallbladder wall. (B) MRCP image reveals a torsion-like defect at the level of the gallbladder neck.

CT imaging revealed significant thickening of the gallbladder wall with edematous changes. Magnetic resonance cholangiopancreatography (MRCP) revealed a torsion-like defect at the gallbladder neck (Figure 3).

**Figure 4:**
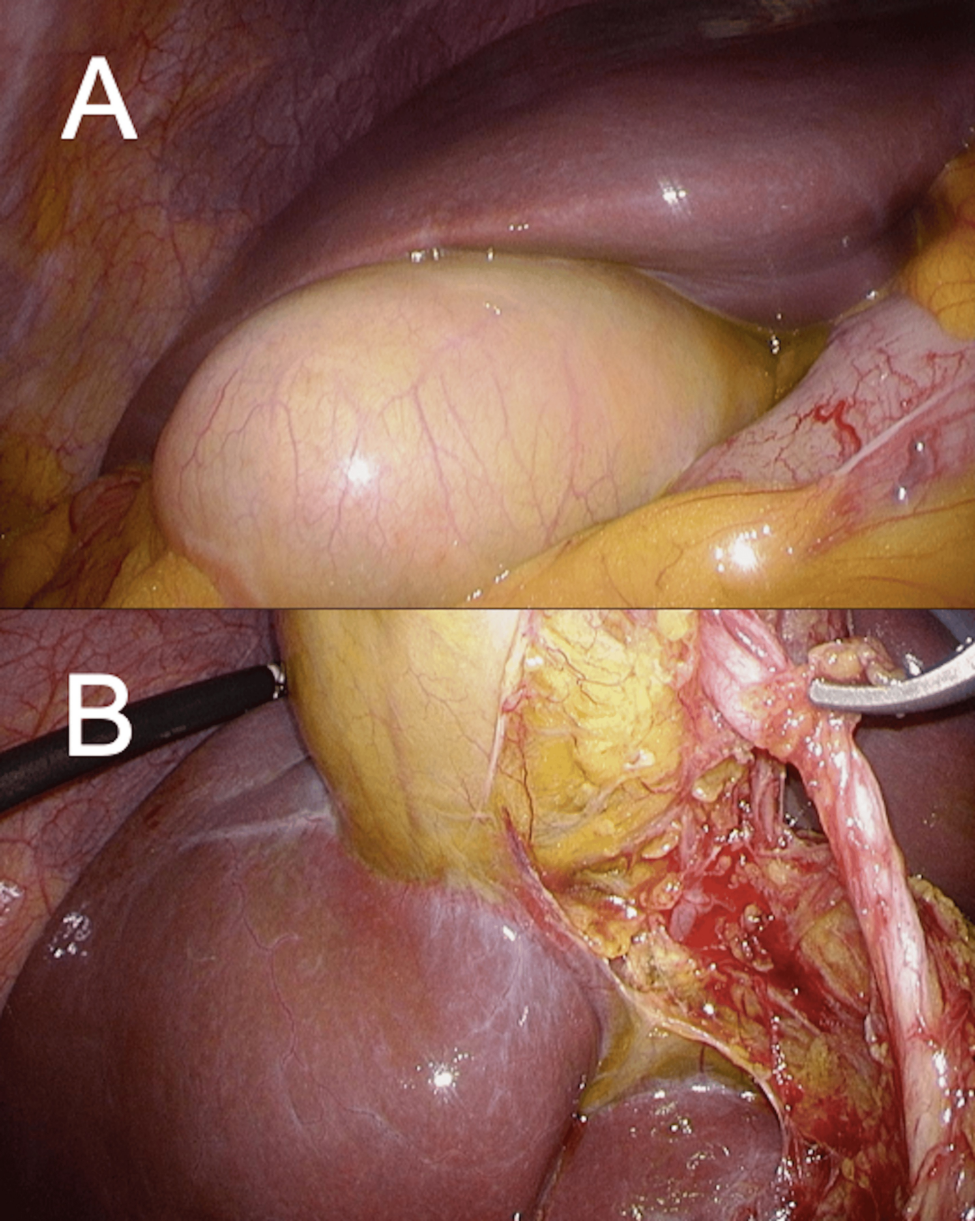
Intraoperative laparoscopic findings of Case 2. (A) Initial laparoscopic view shows an enlarged gallbladder with mild ischemic changes. (B) The view after exploration demonstrates a significantly elongated and twisted cystic duct.

Laparoscopic exploration showed an enlarged gallbladder with mild ischemic changes and a significantly elongated and twisted cystic duct (Figure 4). The condition was classified as Gross type I with incomplete necrosis and 90° torsion.

Laparoscopic cholecystectomy was completed in 59 minutes without conversion to open surgery. The patient recovered smoothly and was discharged on postoperative day 1. At the one-month follow-up, he remained asymptomatic with complete resolution of his preoperative symptoms.

Table 3 summarizes the key clinical features, surgical findings, and outcomes of both cases, highlighting the distinct presentations and degrees of torsion while demonstrating successful laparoscopic management in both scenarios with comparable operative times despite different levels of anatomical complexity.

**Table 3:** Clinical characteristics and surgical outcomes of two cases of gallbladder volvulus This is the summary of patient demographics, clinical presentations, operative findings, and surgical outcomes in two contrasting cases of gallbladder volvulus. Case 1 represents an acute presentation with Gross type II complete torsion in an elderly female, while Case 2 demonstrates a subacute presentation with Gross type I incomplete torsion in a middle-aged male. Both cases were successfully managed with Lap-C with comparable operative times. The degree of torsion shows correlation with the severity of necrosis and urgency of surgical intervention. Lap-C: Laparoscopic Cholecystectomy

Patient	1	2
Age, Sex	97, female	52, male
Symptoms	Epigastric pain	Right hypochondrial pain (position dependent)
Gross classification	Type II	Type I
Degree of necrosis	Complete necrosis	Incomplete necrosis
Degree of torsion	270°	90°
Surgical method	Lap-C	Lap-C
Surgery time	69 minutes	59 minutes

## Discussion

Gallbladder volvulus is a rare surgical emergency that requires prompt recognition and intervention. The two cases presented here demonstrate distinct clinical manifestations and pathogenic mechanisms, highlighting the spectrum of presentations surgeons may encounter.

Epidemiologically, gallbladder volvulus shows a strong predilection for elderly females, as seen in our first case of a 97-year-old female. However, our second case, featuring a 52-year-old male, underscores that this condition can affect patients across different age groups and genders, albeit less commonly.

Several anatomical and physiological factors contribute to the development of gallbladder volvulus. The presence of a floating gallbladder, characterized by a free mesentery, is considered a prerequisite for torsion. Risk factors include both congenital and acquired conditions, such as anatomical variations in mesenteric attachment, visceroptosis, and postural kyphosis [4].

The degree of torsion appears to correlate with the severity of presentation and extent of ischemic changes. In our cases, the patient with 270° torsion (Case 1) presented with acute symptoms and complete necrosis, necessitating emergency surgery. Conversely, the patient with 90° torsion (Case 2) experienced a subacute course with incomplete necrosis, allowing for semi-emergency surgical intervention. This finding emphasizes the importance of early recognition and intervention to prevent progression to severe ischemia and necrosis.

Diagnostic imaging plays a critical role in preoperative evaluation, though accurate diagnosis remains challenging due to the variable clinical presentation. While ultrasonography (US) is often the initial imaging modality, CT and MRCP provide valuable additional diagnostic information [6,7]. In our second case, MRCP was instrumental in identifying a torsion-like defect at the gallbladder neck, which facilitated preoperative planning [8].

**Figure 5:**
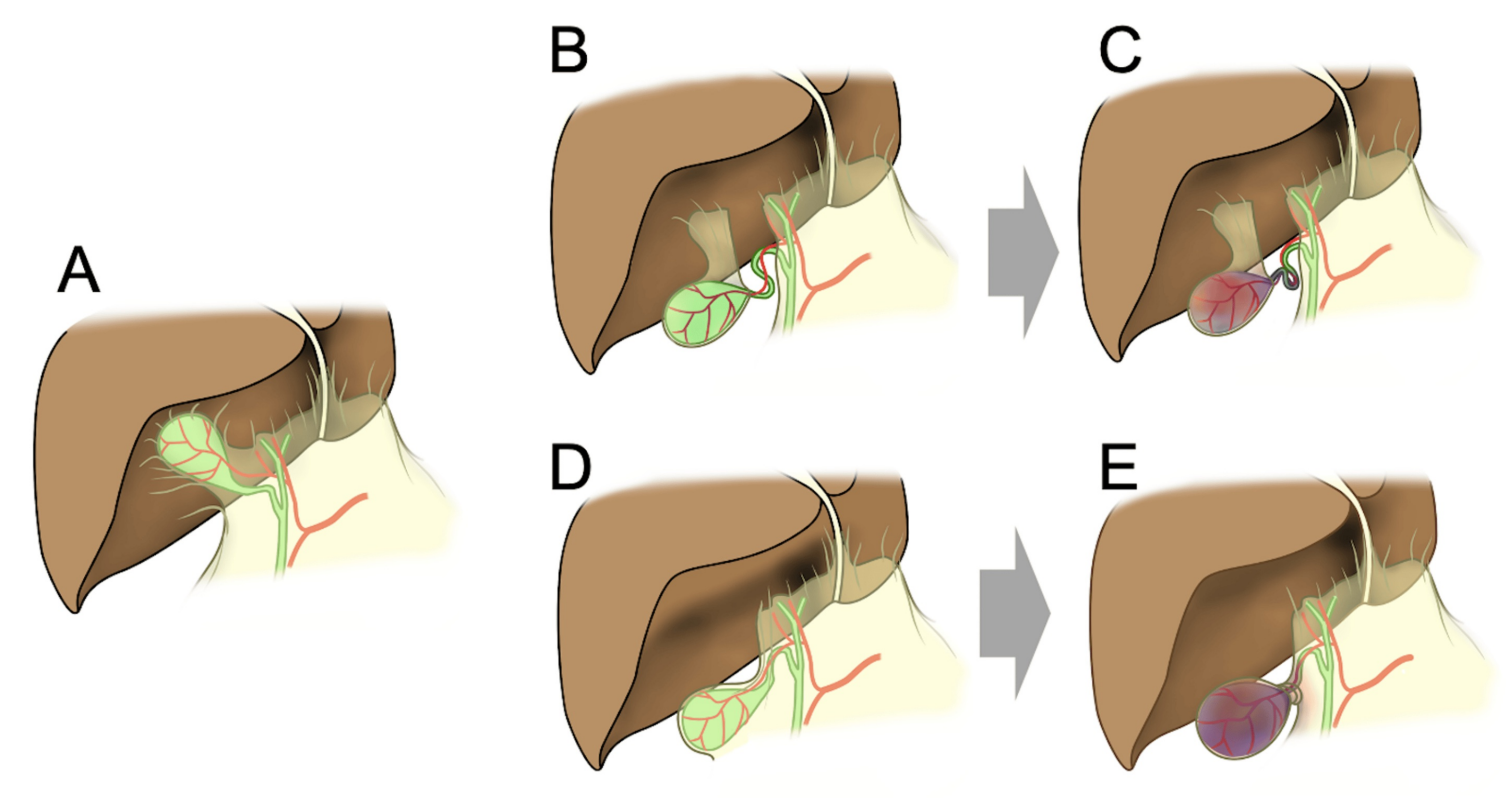
Schematic illustrations of gallbladder anatomy and changes in gallbladder volvulus (A) Normal gallbladder anatomy with typical hepatic attachment (B) Gross type I volvulus showing incomplete torsion and minimal ischemic changes (C) Representative findings of type I volvulus, demonstrating cystic duct bending and incomplete rotation requiring semi-emergency intervention (D) Gross type II volvulus showing complete torsion with severe ischemic changes, necessitating emergency surgical intervention (E) Treatment algorithm based on the Gross classification and degree of necrosis Image credit: Naomi Takagi, Medical Illustrator (Japan)

The Gross classification system provides a useful framework for understanding anatomical variations in gallbladder volvulus [9]. Type I cases, as seen in our male patient, typically present with incomplete torsion and a less acute course. In contrast, type II cases, exemplified by our female patient, often involve complete torsion and severe ischemic changes, requiring urgent surgical intervention. Understanding these classifications aids in tailoring management strategies based on the degree of torsion and necrosis (Figure 5).

**Figure 6:**
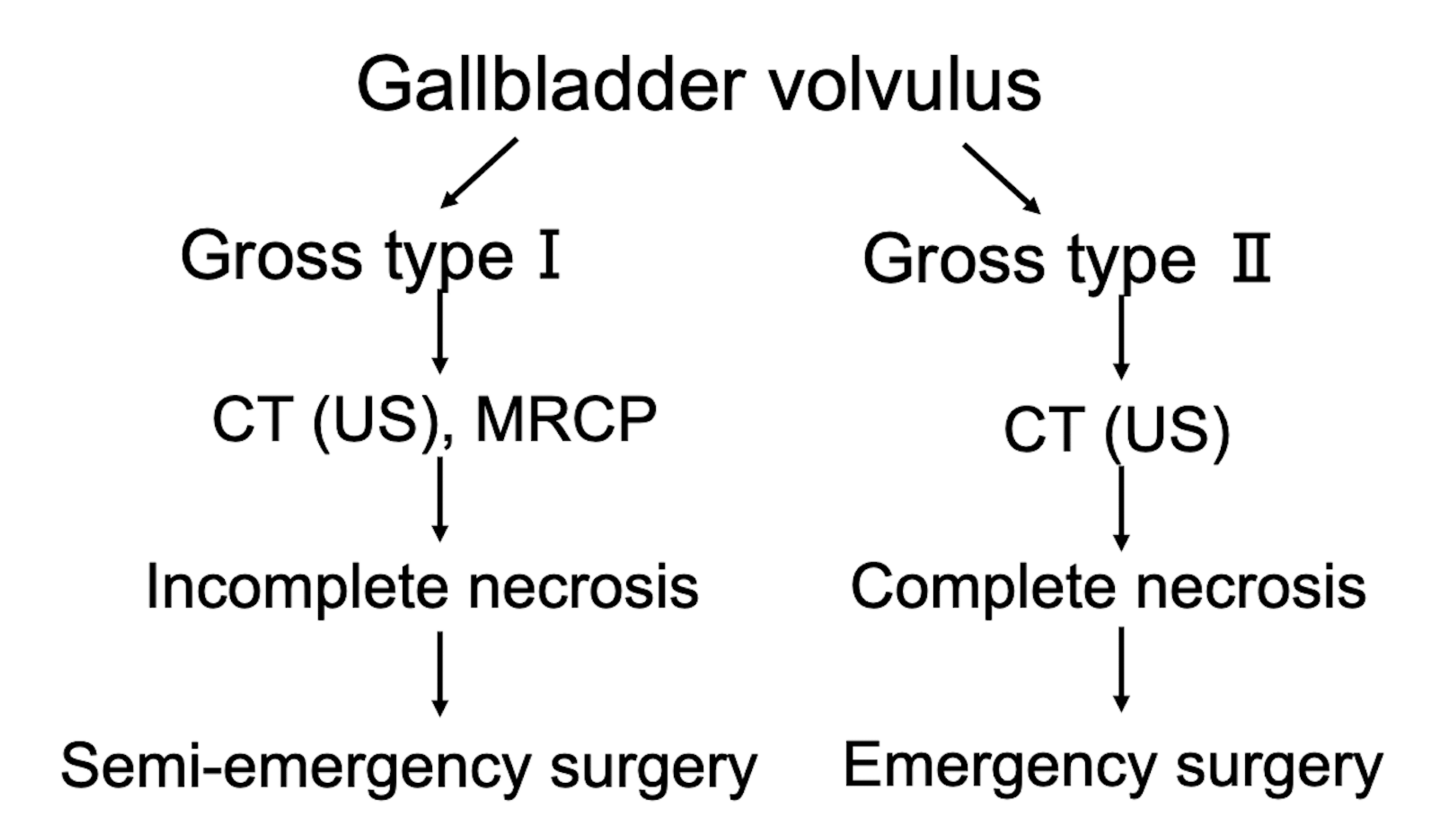
Diagnostic and treatment algorithm for gallbladder volvulus This is the management strategy guided by Gross classification and severity of necrosis. Gross type I volvulus typically requires semi-emergency surgery, while Gross type II volvulus necessitates emergency intervention due to severe ischemic changes. Appropriate imaging studies (CT/US for type II, additional MRCP for type I) assist in preoperative diagnosis and surgical planning. US: Ultrasonography, MRCP: Magnetic Resonance Cholangiopancreatography

The diagnostic and treatment algorithm depicted in Figure 6 illustrates our approach to managing gallbladder volvulus based on the Gross classification.

Type I cases typically present with incomplete torsion and allow time for more extensive imaging workup including MRCP, while type II cases require immediate surgical intervention based on CT/US findings alone due to complete necrosis. This algorithm emphasizes the correlation between the degree of torsion and the urgency of surgical intervention, with type II cases requiring emergency surgery and type I cases amenable to semi-emergency intervention. This systematic approach helps optimize timing of surgical intervention while ensuring appropriate preoperative evaluation.

Laparoscopic cholecystectomy remains the standard treatment for gallbladder volvulus and has been associated with successful outcomes in both acute and subacute presentations [10]. In our experience, careful port placement, gentle handling of the twisted gallbladder, and meticulous dissection of critical structures are key to achieving successful laparoscopic management. Operative times in our cases were 69 and 59 minutes, demonstrating the feasibility of the laparoscopic approach in different clinical scenarios.

This report has certain limitations. As a two-case study, the findings may not be generalizable to all presentations of gallbladder volvulus. Additionally, the retrospective nature of our literature review introduces potential selection bias in the outcomes and complications reported.

Future research should aim to establish standardized diagnostic criteria and evidence-based guidelines for the timing of surgical intervention. Prospective studies are warranted to explore the role of preventive surgery in high-risk patients with floating gallbladder anatomy.

## Conclusions

This report emphasizes the spectrum of gallbladder volvulus presentations and the critical importance of early recognition. Our analysis suggests that the degree of torsion correlates with the acuity of presentation and extent of tissue ischemia, influencing the timing of surgical intervention. The contrasting cases highlight that while gallbladder volvulus predominantly affects elderly females, it can occur across different age groups and genders. Our experience supports the efficacy of laparoscopic cholecystectomy as the standard surgical approach in both acute and subacute presentations, with appropriate imaging selection based on the Gross classification system playing a crucial role in preoperative planning.
